# Affinity for Poetry and Aesthetic Appreciation of Joyful and Sad Poems

**DOI:** 10.3389/fpsyg.2016.02051

**Published:** 2017-01-10

**Authors:** Maria Kraxenberger, Winfried Menninghaus

**Affiliations:** Department for Language and Literature, Max Planck Institute (MPI) for Empirical AestheticsFrankfurt am Main, Germany

**Keywords:** poetry, joy, sadness, liking, beauty, affinity, aesthetic appreciation

## Abstract

Artworks with sad and affectively negative content have repeatedly been reported to elicit positive aesthetic appreciation. This topic has received much attention both in the history of poetics and aesthetics as well as in recent studies on sad films and sad music. However, poetry and aesthetic evaluations of joyful and sad poetry have received only little attention in empirical studies to date. We collected beauty and liking ratings for 24 sad and 24 joyful poems from 128 participants. Following previous studies, we computed an integrated measure for overall aesthetic appreciation based on the beauty and liking ratings to test for differences in appreciation between joyful and sad poems. Further, we tested whether readers' judgments are related to their affinity for poetry. Results show that sad poems are rated significantly higher for aesthetic appreciation than joyful poems, and that aesthetic appreciation is influenced by the participants' affinity for poetry.

## Introduction

Many, if not most, poems are “sad” in terms of their emotional content, with their artistic construction (word choice, prosody) also expressing feelings of sadness, loss, and despair. Paul Celan's *Death Fugue*, Walt Whitman's *O Captain! My Captain!* and W.H. Auden's *Funeral Blues* are only three of the myriad examples for this. Importantly, readers do not just cognitively decode the emotional context and decipher the emotional expression of poems, but apparently also genuinely feel the sadness by way of empathy, emotional contagion, identification, or other means of emotional transfer (Lundqvist et al., 2009; Gerger et al., 2014). However, is sadness not an emotion we prefer *not* to feel? Or do we appreciate sadness in aesthetic contexts, such as reading poetry, as something positive? And do we appreciate happier, more joyous poems less than sad poems, however paradoxical this may seem?

Intuitively, positive aesthetic evaluation and the emotional classification of artworks as joyful or affectively positive seem very closely related. However, movies, music, and poems with sad, i.e., affectively negative, content have repeatedly been reported to be highly appreciated aesthetically. Notably, a rating study of the perception of sad and joyful music excerpts found a significant positive correlation between perceived sadness and perceived beauty (Eerola and Vuoskoski, 2011). Likewise, Oliver and Bartsch (2011, p. 31) suggested that the “experience of appreciation is often thought to be tied more closely with sad than joyful affect.”

Throughout the history of poetics and aesthetics, philosophers and poets have tried to tackle the question of why people enjoy and appreciate feelings of sadness (e.g., Hume, 1757/1793; Schiller, 1792/2006). Hanich et al. (2014) suggested that the overall positive feeling of *being moved* can be understood as a cause of the pleasure associated with negative emotions expressed in or elicited by sadly moving films (for similar findings, see Wassiliwizky et al., 2015). Other mediator emotions that appear to have the power to integrate feelings of sadness into an overall pleasurable emotional trajectory are feelings of nostalgia (Sedikides et al., 2008), tenderness, peacefulness, and relaxation (Taruffi and Koelsch, 2014).

The enjoyment of negative emotions in art reception has also been shown to be influenced by individual differences regarding tendencies to experience states of absorption and music-elicited empathy (Garrido and Schubert, 2011; Taruffi and Koelsch, 2014). Subjectivist theories understand aesthetic evaluation to be mainly determined by individual differences in prior experiences and personal attitudes (e.g., Dewey, 1934/2005). Since frequency measures of exposure to literature and scales like the Author-Recognition-Test (ART; Stanovich and West, 1989; Aacheson et al., 2008) focus mainly on narratives, they are of little use for assessing exposure to or familiarity with the genre of poetry. We here consider readers' general affinity for poetry (see below) as a trait variable that may influence their appreciation of given poems.

Aesthetic judgments, such as those of liking and beauty are often correlated (Brattico et al., 2011; Lüdtke et al., 2014) and understood to be closely related (cf. Reber et al., 2004). However, this is by no means always the case. For instance, horror films are clearly liked by their customary viewers, but research on horror films has not reported any strong experiences of beauty in this context; rather, liking appears to be driven primarily by high affective arousal, thrills, and suspense (Sparks and Ogles, 1994; Hoffner and Levine, 2005; Andrade and Cohen, 2007; Robinson et al., 2014). Similarly, artworks can be liked for being interesting, shocking, a good satire, or even for being markedly ugly (Schlegel, 1795–1797/1979; Rosenkranz, 1853/2015). In such cases, attributions of beauty are apparently no prerequisite for liking. In fact, the partial separation of perceived aesthetic appeal from beauty is one of the major topics and achievements of later eighteenth century, and specifically of post-Kantian aesthetics (for a programmatic volume of essays on this issue see Jauß, 1991).

However, for all these reasons not to commingle judgments of beauty and aesthetic liking, there is some empirical evidence that suggests a very close association between the two judgments in particular contexts. Sad music is one important example; liking of sad music routinely coincides with perceiving high degrees of beauty (Eerola and Vuoskoski, 2011; Taruffi and Koelsch, 2014). Regarding poetry, beauty has been shown—in pronounced contrast to novels and plays—to be (still) the prime expectation of perceived aesthetic appeal among non-professional contemporary readers, regardless of the key emotional tonality (Knoop et al., 2016). A recent experimental study has shown—with a specific focus on the role of parallelistic diction—that liking judgments and beauty attributions for poetry correlate positively both with each other and with self-reported feelings of joy, sadness and being moved (Menninghaus et al., 2016). In light of these data, we decided to follow previous studies on both music and literature (Brattico et al., 2011; Lüdtke et al., 2014) in measuring perceived overall aesthetic appeal by using the average of beauty and liking ratings as a composite index for aesthetic appreciation.

To date, no empirical investigation has considered aesthetic evaluation(s) of poetry in light of the respective poems' emotional classification and of readers' affinity for poetry. We set out to do precisely this. We expected higher aesthetic appreciation for sad poems than for joyful ones. Further, we hypothesized readers' self-reported affinity for poetry to be positively related to their aesthetic evaluations.

## Methods

### Corpus

We compiled a corpus of 48 German poems that comprises 24 joyful poems and 24 sad poems. The poems were written, or published for the first time, by 39 authors between 1828 and 1978, vary substantially in length, and include both rhymed and metered and non-rhymed and non-metered poems (for details see Table 1). Since most of these poems were published in a well-known anthology (Reschke, 1992; cf. Gernhardt, 2012), our sample of poems may well be representative. We based our a priori classification of the poems as either joyful or sad on phenomenological descriptions of joy and sadness (cf. Schmitz, 1969; Demmerling and Landweer, 2007) and the poems' main themes (cf. Kraxenberger and Menninghaus, 2016).

**Table 1:** **Titles, Authors, Publication Date, General Features, and Mean-Emotion Ratings of the Analyzed Poems**.

**Title**	**Author**	**Publication date**	**No of lines**	**No of stanzas**	**No of words**	**End-rhymed**	**Consistent meter**	**Joyful vs sad**.	**Emotion-rating**
*Tristesse*	Benn, Gottfried	1956	16	4	113	yes	yes	sad	5.69
*Sommersonett*	Bergengruen, Werner	1950	14	4	84	yes	yes	joy	1.94
*Novemberabend*	Boldt, Paul	1912	8	2	45	yes	yes	sad	5.38
*Der Kuss*	Borchert, Wolfgang	1946	12	3	84	yes	yes	joy	3.38
*Doppelte Freude*	Busch, Wilhelm	1909	8	1	48	yes	yes	joy	2.38
*Rückkehr*	Cordan (Horn), Wolfgang	1951	12	3	67	yes	no	sad	6.38
*Blick ins Licht*	Dehmel, Richard	1913	21	4	101	yes	yes	joy	3.56
*Fähre Schenkenschanz*	Delius, Friedrich Christian	1981	8	2	68	yes	no	joy	3.69
*Sterben*	Ehrenstein, Albert	1961	13	3	63	no	no	sad	5.50
*Heimkehr*	Ehrenstein, Albert	1961	12	1	58	yes	no	sad	5.81
*Call it love*	Enzensberger, Hans Magnus	1957	16	1	87	no	no	joy	3.06
*april*	Enzensberger, Hans Magnus	1963	23	4	99	no	no	sad	2.56
*trennung*	Enzensberger, Hans Magnus	1957	18	3	79	no	no	joy	5.88
*Freundliche Nähe*	Ernst, Otto	1917	16	3	95	yes	yes	joy	2.00
*Schön und gut und klar und wahr*	Gernhardt, Robert	1990	12	5	87	yes	no	joy	3.25
*Trauermarsch*	Goll, Yvan	1960	13	1	88	yes	yes	sad	6.69
*O leuchtender Septembertag*	Haller, Paul	1922	12	3	64	yes	yes	joy	2.38
*Spät*	Hardekopf, Ferdinand	1963	12	3	74	yes	yes	sad	6.31
*Regen*	Hatzfeld, Adolf von	1919	12	3	86	yes	yes	sad	5.63
*Schwermut*	Henckell, Karl	1921	24	1	141	yes	yes	sad	6.25
*Im Nebel*	Hesse, Hermann	1905	16	1	76	yes	yes	sad	6.19
*Fröhlichkeit*	Heym, Georg	1911	12	3	81	yes	yes	joy	2.25
*Letzte Wache*	Heym, Georg	1964	16	3	82	yes	yes	sad	6.88
*Nicht alle Schmerzen*	Huch, Ricarda	1971	12	3	71	yes	no	sad	5.75
*Das berühmte Gefühl*	Kaléko, Mascha	1978	14	3	89	yes	yes	sad	5.38
*Traurigkeit*	Kalkowska, Eleonore	1916	10	5	42	yes	no	sad	6.00
*Das Glück im Spiel*	Klabund	1927	14	4	104	yes	yes	joy	3.50
*Liebeslied: Dein Mund*	Klabund	1927	16	1	84	yes	yes	joy	2.63
*Erfüllung*	Klemm, Wilhelm	1919	12	3	93	yes	no	joy	1.89
*Freude*	Krzyzanowski, Otfried	1919	4	1	30	no	no	joy	2.50
*Dämmerung*	Lasker-Schüler, Else	1943	10	1	64	yes	yes	sad	5.50
*Liebeslied*	Lichtenstein, Alfred	1919	6	1	36	no	no	joy	1.94
*Der Rauch auf dem Felde*	Lichtenstein, Alfred	1914	25	7	110	no	yes	sad	6.13
*Nachtmusik*	Loerke, Oskar	1958	12	1	46	yes	no	sad	5.38
*Radfahrt*	Malkowski, Rainer	1977	14	1	35	yes	no	joy	2.63
*Licht ist Liebe*	Morgenstern, Christian	1914	12	4	54	yes	yes	joy	3.44
*Das ästhetische Wiesel*	Morgenstern, Christian	1905	11	4	30	yes	no	joy	2.50
*Die Windhosen*	Morgenstern, Christian	1910	12	3	60	yes	yes	joy	3.63
*Er ist's*	Mörike, Eduard	1828	10	1	39	yes	yes	joy	1.50
*Vereinsamt*	Nietzsche; Friedrich	1882	23	6	113	yes	yes	sad	6.06
*Das Leben ist gut und licht*	Rilke; Rainer Maria	1913	8	2	51	yes	yes	joy	2.44
*Morgenwonne*	Ringelnatz, Joachim	1933	12	3	57	yes	yes	joy	1.63
*Nach derTrennung: Lichterfelde*	Ringelnatz, Joachim	1929	20	4	114	yes	yes	sad	5.38
*Elegie*	Schwachhofer, René	1964	13	3	56	yes	no	sad	6.63
*Pans Trauer*	Stadler, Ernst	1911	14	1	165	yes	yes	sad	5.25
*Das Licht*	Strub, Urs Martin	1946	9	1	47	yes	yes	joy	3.19
*Die Zerwartung*	Thoor, Jesse	1965	14	4	112	yes	yes	sad	6.13
*Ostersamstag*	Wagner, Christian	1890	20	5	93	yes	yes	sad	6.25
Mean (SD)			13.60 (4.58)	2.77 (1.51)	76.35 (28.87)				

We expected the selected poems to be easy to comprehend, because they do not include words, metaphors and sentences that are particularly rare or difficult to understand. We controlled for differences between joyful and sad poems by applying several analyses of variance (ANOVAs) or, in the case of nominal variables, Chi-Square tests. Results showed no significant association between the classification of the poems as joyful or sad and the occurrence of end rhymes (*X*^2^ (1, *N* = 48) = 0.17; *p* = 0.68). Further, results showed no differences between joyful and sad poems regarding their metrical structure (metrically bound vs. free verse; *X*^2^ (1, *N* = 48) = 0.09; *p* = 0.76), or their organization in stanzas (*X*^2^ (1, *N* = 48) = 0.10; *p* = 0.76). Poems classified as joyful showed no differences when compared to those poems that were classified as sad in terms of number of stanzas per poem (joyful poems: *M* = 2.54; *SD* = 1.29; sad poems: *M* = 3.00; *SD* = 1.69; *p* = 0.30). However, the sad poems tend to have a few more words (*M* = 86.13; *SD* = 30.75) and lines (*M* = 15.17; *SD* = 4.80) than the joyful poems (*M*_words_ = 66.58; *SD*_words_ = 23.63; *p*_words_ = 0.02; *M*_lines_ = 12.04; *SD*_lines_ = 3.84; *p*_lines_ = 0.02).

### Participants

One hundred and twenty-eight participants (84 women, 44 men) took part in the rating study. The mean age was 24.5 years (*SD* = 4.36, *min* = 18, *max* = 37). Inclusion criteria for study participation were having German as (one) native language and being of full legal age. All participants gave their informed consent and received monetary compensation or course credit.

### Procedure and questionnaire

Participants were instructed to silently read each poem twice, in a calm and attentive manner. This instruction was used because previous studies employing a rereading paradigm suggest that the effects of literary language consolidate over time and that repeated reading supports a greater “depth of appreciation” (Dixon et al., 1993, p. 17; see also Hakemulder, 2004) and should enhance participants' comprehension. After the second reading, participants rated the poems on several items, using a pen and paper questionnaire^1^.

Our questionnaire included a rating item (hereafter: Emotion) to measure whether participants assigned the perceived emotional tonality of the respective poems rather to the pole of joy (1) or to that of sadness (7). In order to evaluate participants' aesthetic appreciation, we used two separate items (*How beautifully is the poem written?* (Beauty) and *How much do you like this poem?* (Liking), with both items ranging from 1 (not at all) to 7 (very much). As reported in the Introduction, we derived an integrated measure for overall Aesthetic Appreciation from these two ratings. We did so by averaging the ratings for Liking and Beauty. This pooled index for Aesthetic Appreciation had a high level of internal consistency, as determined by a Cronbach's alpha of 0.9.

Finally, participants were asked to indicate whether they knew the respective poems and to report their age (in years), gender, and affinity (hereafter: Affinity) for poetry by stating to what extent they generally enjoy reading or listening to poetry, ranging from 1 (not at all) to 7 (very much).

Given the size of the corpus, we opted for a between-participants design with the intention of reducing possible fatigue and carryover effects by presenting only a few stimuli per participant. In order to keep the survey short, the 48 poems were divided into eight groups of six poems each. Each poem received 16 ratings, and each participant read and rated six poems—three joyful and three sad ones. The sequence of the rating items and the order of presented poems were randomized between participants.

## Results^2^

To test whether participants confirmed our pre-classification of the poems as either joyful or sad, we inspected the mean values of all poems on the item Emotion. The means of the poems that were pre-classified as joyful (*M* = 2.72, *SD* = 0.65, *min* = 1.63, *max* = 3.69) were all below the midpoint of the scale (4), whereas the means of the poems that were pre-classified as sad (*M* = 5.93, *SD* = 0.46, *min* = 5.25, *max* = 6.88) were all above the midpoint (for mean-Emotion ratings, see Table 1). To control for possible effects of participants' familiarity with the poems, we excluded two joyful poems that were familiar to more than 10% of the participants from further analyses^3^. On average, participants indicated an affinity of 5.05 for reading or listening to poetry (*SD* = 1.58, *min* = 1, *max* = 7).

Using the emotional pre-classification of the poems (coded in a binary way: 1 (joyful) vs. −1 (sad)), participants' Affinity and the number of words per poem as independent variables, we applied a linear mixed effects analysis with which we predicted Aesthetic Appreciation (as defined above). We also included intercepts for participants and poems as random effects in this analysis (Baayen et al., 2008).

Results show a significant effect of the emotional classification (*t* = −2.24; *p* = 0.03) and a significant effect of participants' Affinity for poetry (*t* = 3.45; *p* ≤ 0.001) on Aesthetic Appreciation. The number of words per poem was unrelated to participants' ratings (*t* = 0.72; *p* = 0.47; see Table 2 for estimates and standard errors of fixed effects and the intercept).

**Table 2:** **Fixed effects for the model predicting Aesthetic Appreciation**.

**Parameter**	**b (SE)**
Intercept	3.97 (0.18)^***^
Emotional Category	−0.17 (0.08)^*^
Affinity	0.14 (0.04)^***^
Word number per poem	0.002 (0.003) n.s.

Estimates and standard errors (in parentheses). The applied model had the following syntax in R: Aesthetic Appreciation ~ Emotional Category + Affinity + (1|Participant) + (1|Poem); n.s.: p > 0.05;

* p < 0.05;

*** *p ≤ 0.001*.

**Figure 1:**
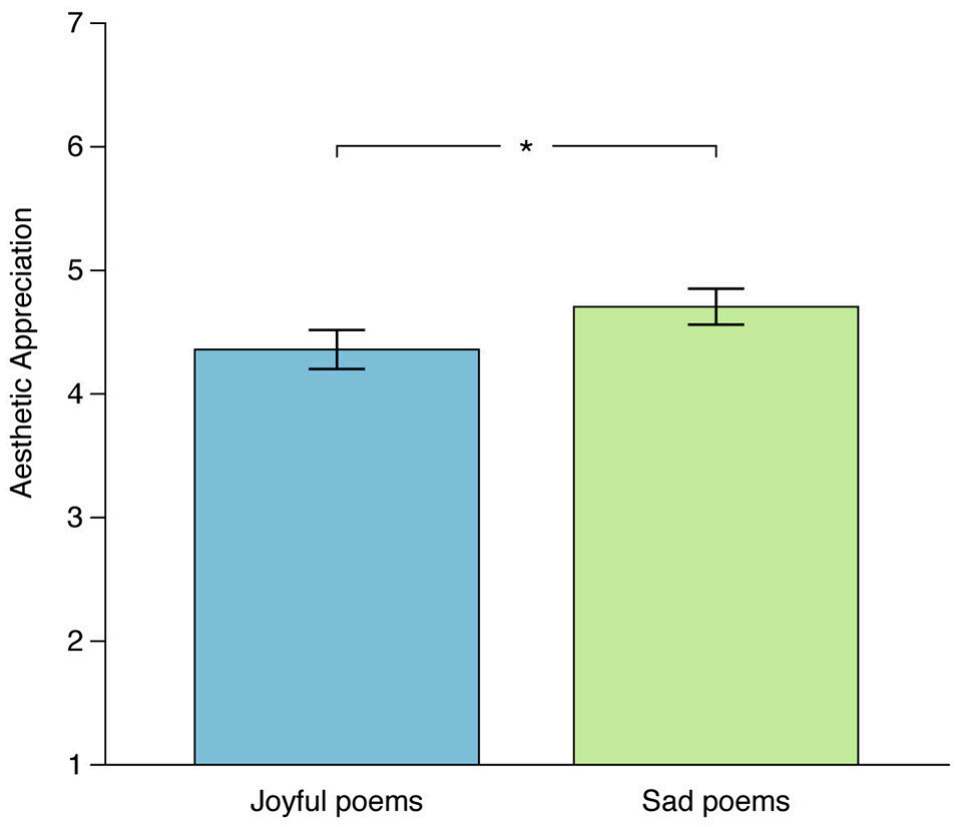
**Boxplots showing mean values of the used averaged values of Liking and Beauty ratings (Aesthetic Appreciation), separately displayed for joyful and sad poems**. ^*^p < 0.05.

An inspection of the mean values for Aesthetic Appreciation showed that sad poems were rated higher (*M* = 4.70, *SD* = 1.45, *n* = 24) than joyful poems (*M* = 4.36 *SD* = 1.51, *n* = 22; see Figure 1).

## Discussion and outlook

Our analyses show that Affinity for poetry clearly affects ratings of Aesthetic Appreciation. Results also show a significantly higher Aesthetic Appreciation for sad than for joyful poems.

With all due caution, our findings can be interpreted as supporting theories of pleasure in negative affect that suggest a positive relation between sad stimuli and aesthetic appreciation (cf. Taruffi and Koelsch, 2014). Considering the well-known effect of familiarity on aesthetic evaluation (cf. Zajonc, 1968; Calvo-Merino et al., 2008), an explanation for our finding of higher Aesthetic Appreciation for sad poems could be that sad poems simply constitute a greater share of the (Western) tradition of poetry than joyful poems. This higher familiarity with sad poems might be the reason why they are generally more appreciated than joyful poems.

The results presented here are certainly limited by the chosen corpus, as well as the personal and textual variables that were analyzed. Therefore, they could be complemented by follow-up studies that include additional situational factors, incorporate a broader exploration of readers' characteristics, and do not exclusively rely on behavioral data.

Furthermore, due to the theoretical separation between beauty and other forms of appreciation within the realm of the arts, future studies exploring empirically possible differences between judgements of liking and beauty are called for. Future investigations on this topic should consider co-occurrence patterns of different linguistic concepts that might reflect different mental constructs by applying corpus-linguistic or qualitative approaches. In addition, such future studies should also aim at explaining a differentiation between different forms of aesthetic appreciation by integrating psychological models of aesthetic appreciation and experience as well as an explanation of the underlying psychological processes (for a review on current psychological models of art experience for the visual arts, see for example Pelowski et al., 2016; see also Jacobs et al., 2016, this issue). Whether poetry is the appropriate genre for differentiating judgments of beauty and liking is, however, an open research question. Alternatively, other literary genres of fictions or media forms, such as movies that foster an involvement of readers and viewers with the expression of ugliness, disgust and horror might be more prone for a differentiation of different forms of positive evaluations.

Summing up, our study indicates that sad poetry indeed is appreciated more than joyful poetry. Furthermore, the higher our affinity to poetry in general, the higher our positive evaluations tend to be, independent of a poem's emotional content.

## Ethics statement

All experimental procedures were ethically approved by the Ethics Council of the Max Planck Society, and were undertaken with informed consent of each participant. Human participants read and rated six German poems each. No vulnerable populations were involved.

## Author contributions

MK and WM jointly designed the study and interpreted the data. MK compiled the poetic corpus, gathered behavioral data, conducted data analyses and wrote the paper.

## Funding

Data acquisition for this paper was made possible through the support of the Research Cluster Languages of Emotion (EXC302), which was funded by the German Research Association DFG and hosted by the Freie Universität Berlin. The writing and data analysis was conducted at the Max Planck Institute for Empirical Aesthetic in Frankfurt am Main, Germany.

### Conflict of interest statement

The authors declare that the research was conducted in the absence of any commercial or financial relationships that could be construed as a potential conflict of interest.

## Footnotes

^1^Given the hypotheses and research questions on which we focus in this study, some further items from the questionnaire were not considered in the analyses presented here. We have reported all other results in a separate study that, complementary to the one presented in this article, includes neither the beauty nor the liking ratings (cf. Kraxenberger and Menninghaus, 2016).

^2^All analyses, apart from the linear mixed effects analyses reported below, were conducted in SPSS (IBM SPSS Statistics for Windows, Version 22.0, IBM Corp, 2013). A visual inspection of normal Q-Q plots showed that both our behavioral and our phonological data were approximately normally distributed. We used R (R Core Team, 2013) and lme4 (Bates et al., 2014) to perform linear mixed effects analyses. P-values were obtained via lmerTest (Kuznetsova et al., 2015). Apart from the linear mixed effect analyses, our analyses are based on mean values.

^3^*Das ästhetische Wiesel* by C. Morgenstern, known to 4 of its 16 raters (25%) and *Er ist's* by E. Mörike, known to 10 of its 16 raters (62.5%).
